# Supplementary material for: Immunogenicity and Safety of the Third Booster Dose with mRNA-1273 COVID-19 Vaccine after Receiving Two Doses of Inactivated or Viral Vector COVID-19 Vaccine
Source: Vaccines (Basel). 2023 Feb 27;11(3):553. doi: 10.3390/vaccines11030553 (PMC10053262; doi:10.3390/vaccines11030553)
Supplement: Supplementary file 1 [file vaccines-11-00553-s001.zip › vaccines-2171204-supplementary.pdf]

Table S1. Local and systemic reactogenicities within 7 days after mRNA-1273 booster among CoronaVac (SV)-primed and AZD1222 (AZ)-primed.

Side effect	Total<br>N (%)	Post SV (2 doses)<br>N (%)	Post AZ (2doses)<br>N (%)	*p*-value
Fever				
- Grade 1	6 (6.19)	3 (6.38)	3 (6.00)	
- Grade 2	5 (5.15)	4 (8.51)	1 (2.00)	0.298
- Grade 3	2 (2.06)	0 (0.00)	2 (4.00)	
Pain at injected site				
- Grade 1	29 (39.9)	18 (38.30)	11 (22.00)	
- Grade 2	50 (51.55)	20 (42.55)	30 (60.00)	0.135
- Grade 3	7 (7.22)	2 (4.26)	5 (10.00)	
Swelling at injected site				
- Grade 1	14 (14.43)	9 (19.15)	5 (10.00)	0.052
- Grade 2	(5.15) 5	(0.00) 0	5 (10.00)	
- Grade 3	(0.00) 0	(0.00) 0	0 (0.00)	
Redness at injected site				
- Grade 1	(6.19) 6	5 (10.64)	(2.00) 1	0.058
- Grade 2	(3.09) 3	0 (0.00)	(6.00) 3	
- Grade 3	(0.00) 0	(0.00) 0	(0.00) 0	
Headache				
- Grade 1	28 (28.87)	16 (34.04)	12 (24.00)	
- Grade 2	24 (24.74)	10 (21.28)	14 (28.00)	0.575
- Grade 3	1 (1.03)	0 (0.00)	1 (2.00)	
Fatigue				
- Grade 1	34 (35.05)	15 (31.91)	19 (38.00)	
- Grade 2	21 (21.65)	7 (14.89)	14 (28.00)	0.217
- Grade 3	4 (4.12)	2 (4.26)	2 (4.00)	
Myalgia				
- Grade 1	28 (28.87)	15 (31.91)	13 (26.00)	
- Grade 2	34 (35.05)	13 (27.66)	21 (42.00)	0.320
- Grade 3	4 (4.12)	1 (2.13)	3 (6.00)	
Arthralgia				
- Grade 1	6 (6.19)	2 (4.26)	4 (8.00)	
- Grade 2	5 (5.15)	3 (6.38)	2 (4.00)	0.855
- Grade 3	2 (2.06)	1 (2.13)	1 (2.00)	
Vomiting				
- Grade 1	2 (2.06)	1 (2.13)	1 (2.00)	
- Grade 2	2 (2.06)	0 (0.00)	2 (4.00)	0.745
- Grade 3	0 (0.00)	0 (0.00)	0 (0.00)	
Diarrhea				
- Grade 1	6 (6.19)	4 (8.51)	2 (4.00)	
- Grade 2	0 (0.00)	0 (0.00)	0 (0.00)	0.426
- Grade 3	0 (0.00)	0 (0.00)	0 (0.00)	

N = number of subjects. Adverse events grading according to U.S. Department of Health and Human Services F, CBER. Guidance for Industry Toxicity Grading Scale for Healthy Adult and Adolescent Volunteers Enrolled in Preventive Vaccine Clinical Trials September 2007

[Available from: https://www.fda.gov/media/73679/download. Accessed date 30 November 2021].
